# Evaluation of iron deposition in the motor CSTC loop of a Chinese family with paroxysmal kinesigenic dyskinesia using quantitative susceptibility mapping

**DOI:** 10.3389/fneur.2023.1164600

**Published:** 2023-07-06

**Authors:** Fangfang Xie, Ting Mao, Jingyi Tang, Linmei Zhao, Jiuqing Guo, Huashan Lin, Dongcui Wang, Gaofeng Zhou

**Affiliations:** ^1^Department of Radiology, Xiangya Hospital, Central South University, Changsha, China; ^2^Department of Pharmaceutical Diagnosis, GE Healthcare, Changsha, China; ^3^National Clinical Research Center for Geriatric Disorders, Xiangya Hospital, Central South University, Changsha, China

**Keywords:** paroxysmal kinesigenic dyskinesia (PKD), cortico-striatal-thalamo-cortical loop, quantitative susceptibility mapping (QSM), multi-scale dipole inversion, iron deposition

## Abstract

**Introduction:**

Previous studies have revealed structural, functional, and metabolic changes in brain regions inside the cortico-striatal-thalamo-cortical (CSTC) loop in patients with paroxysmal kinesigenic dyskinesia (PKD), whereas no quantitative susceptibility mapping (QSM)-related studies have explored brain iron deposition in these areas.

**Methods:**

A total of eight familial PKD patients and 10 of their healthy family members (normal controls) were recruited and underwent QSM on a 3T magnetic resonance imaging system. Magnetic susceptibility maps were reconstructed using a multi-scale dipole inversion algorithm. Thereafter, we specifically analyzed changes in local mean susceptibility values in cortical regions and subcortical nuclei inside the motor CSTC loop.

**Results:**

Compared with normal controls, PKD patients had altered brain iron levels. In the cortical gray matter area involved with the motor CSTC loop, susceptibility values were generally elevated, especially in the bilateral M1 and PMv regions. In the subcortical nuclei regions involved with the motor CSTC loop, susceptibility values were generally lower, especially in the bilateral substantia nigra regions.

**Conclusion:**

Our results provide new evidence for the neuropathogenesis of PKD and suggest that an imbalance in brain iron levels may play a role in PKD.

## 1. Introduction

Paroxysmal kinesigenic dyskinesia (PKD) is the most common subtype of paroxysmal movement disorder, which is characterized by transient, recurrent attacks of dyskinesia triggered by sudden movement (1). Such attacks may cause short-term loss of mobility and interfere with daily activities such as walking and working, substantially impairing the quality of life (2). PKD is divided into primary and secondary types based on the etiology, with primary PKD being inherited in an autosomal dominant pattern (1). The proline-rich transmembrane protein 2 (PRRT2) gene, located in the peri-centromeric region of chromosome 16, was the first identified cause of the primary type of PKD (3).

PRRT2 is predominantly expressed in the basal ganglia (4), a key node in the cortico-striatal-thalamo-cortical (CSTC) loop. The motor CSTC loop originates from the motor cortex and is a neuronal circuit that controls movement selection and initiation, reinforcement, and reward (5). The abnormal function of the basal ganglia in PKD has been confirmed by imaging studies. Shirane et al. (6) reported enhanced ictal blood perfusion in the left thalamus detected by single-photon emission computed tomography (SPECT). Subsequent neuroimaging studies discovered that structural and functional abnormalities also exist in the thalamus and cortex of the motor CSTC loop outside the basal ganglia in patients with PKD (7). Kim et al. (8) reported reduced gray matter volume of the bilateral thalami accompanied by shape deformation. In addition, Zhou et al. (9) reported that compared to controls, those with PKD had significantly higher fractional anisotropy values in the right thalamus and lower mean diffusivity values in the left. Moreover, Long et al. (10) found that PKD patients had increased structural white matter fiber tract connectivity between the ventral lateral/anterior thalamic nuclei and the lateral motor cortical area compared to the controls. Zhou et al. (11) and Luo et al. (12) performed voxel-based analyses on the amplitude of low-frequency fluctuation (ALFF) calculated from resting-state functional magnetic resonance imaging (fMRI) data and found that those with PKD had a significant increase in neuronal activity in the putamen and right postcentral gyrus. Additional studies indicated that PKD patients had increased functional connectivity between the ventral lateral/anterior thalamic nuclei and the lateral motor cortical area (10). Further functional network analysis of the small-world properties of the topological organization showed that PKD patients had increased nodal centralities in the left precentral gyrus, basal ganglia, and limbic regions (13). Therefore, the motor CSTC loop where basal ganglia are located was considered to be implicated in the neuropathological mechanism of PKD.

The protein encoded by PRRT2 functions to delay the transition of the voltage-gated sodium channels from an inactivated to a normal resting state. Its deficiency in neurons could therefore accelerate the recovery of sodium channels from an inactivated state, leading to enhanced release of excitatory factors, such as potassium ions or glutamate neurotransmitters (4). Both the anabolism and catabolism of neurotransmitters require iron-containing enzymes and iron-dependent proteins (14). Considering that the abnormal release of neurotransmitters is related to the neural mechanism of PKD (15), iron is required for several neuronal-specific functions such as dopaminergic neurotransmitter synthesis (16), and the motor CSTC loop is speculated to be involved in the pathogenesis of PKD, alterations in iron deposition inside the motor CSTC loop may therefore be expected.

Quantitative susceptibility mapping (QSM) is an emerging MRI technology that is sensitive to detect alterations in magnetic susceptibility by utilizing gradient-echo sequences (17). It has a great potential to map the perturbations of iron distributions in neurological diseases including those with clinical manifestations of movement disorders (18, 19), such as Parkinson's disease (20), Huntington's disease (21), and epilepsy (22–25). However, the spatial distribution of magnetic susceptibility in PKD and its correlation to motor symptoms has not yet been elucidated. To this end, the present study aims to test for the first time the hypothesis that iron homeostasis along the CSTC loop is disrupted in PKD patients.

## 2. Materials and methods

### 2.1. Participants

**Figure 1:**
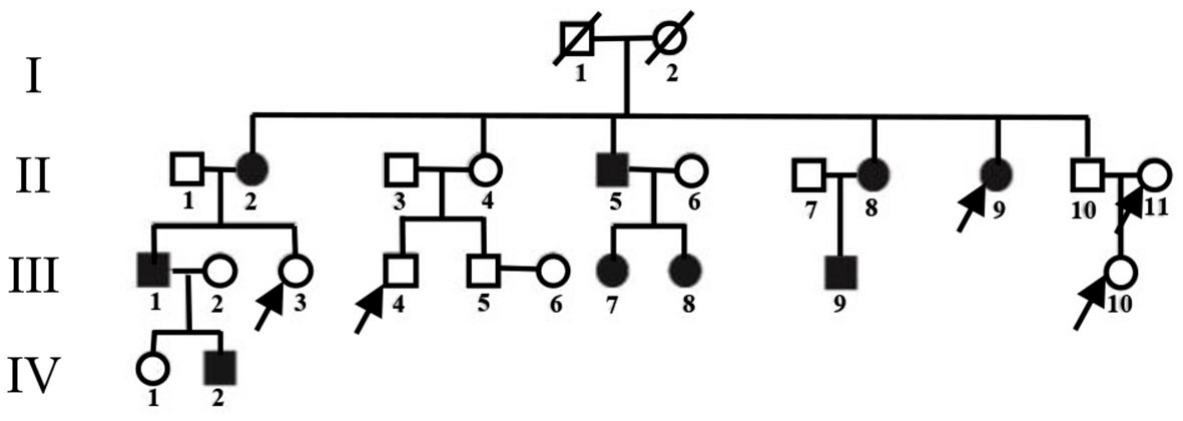
Pedigree of the PKD-affected family. Squares represent males; circles represent females; empty symbols indicate unaffected family members; filled-in symbols indicate individuals with PKD; individuals indicated by arrows were not enrolled.

Study participants were recruited from a four-generation Chinese PKD pedigree (Figure 1), whose clinical manifestations and pathogenic variants have been previously reported (26). A detailed medical history from all available individuals was collected. Thorough physical examinations were performed by two experienced neurologists. The inclusion criteria for the PKD group in the present analysis were (1) meeting the PKD diagnostic criteria established by Cao et al. (27); (2) having PRRT2 gene deletion mutations; (3) having good vision, hearing, and language capacity; and (4) not having any obvious anxiety or depression tendencies. The exclusion criteria were having (1) a history of prior traumatic brain injury, brain tumor, cerebral infarction, intracerebral hemorrhage, epilepsy, or other nervous system disorders and/or (2) MRI scanning incompatibility.

Healthy, close family members of those in the family with PKD were recruited as normal controls (NC). The inclusion criteria were having (1) no PRRT2 gene deletion mutations; (2) good vision, hearing, and language capacity; and (3) no obvious anxiety and/or depression tendencies. The exclusion criteria were the same as above.

The genetic variants were determined by whole-exome sequencing and PCR-Sanger sequencing as described in He et al. (26). Screening for psychological illness was carried out on all participants by two trained neurological physicians using the Symptom Checklist-90-Revised (SCL-90-R), Self-rating Anxiety Scale (SAS), and Self-rating Depression Scale (SDS).

All participants signed informed consent and volunteered to participate. This study was approved by the Ethics Review Committee from Xiangya Hospital of Central South University.

### 2.2. Imaging protocol

Axial QSM images were acquired on a Siemens 3T MRI system (Prisma, Siemens Healthcare, Erlangen, Germany) using a 64-channel head array coil and a multi-echo sequence with the following parameters: repetition time (TR) = 50 ms, flip angle (FA) = 15°, slice thickness (ST) = 1.2 mm, field of view (FOV) = 220 × 192 mm^2^, matrix size = 288 × 252, echo train length (ETL) = 8, echo time (TE) = 10.79 ms, 17.25 ms, 21.35 ms, 25.45 ms, 29.55 ms, 33.65 ms, 37.75 ms, and 41.85 ms, receiver bandwidth (BW) = 260 Hz/Pixel, number of excitations (NEX) = 1, and total acquisition time (TA) = 10 m 37 s.

### 2.3. QSM reconstruction and spatial standardization

**Figure 2:**
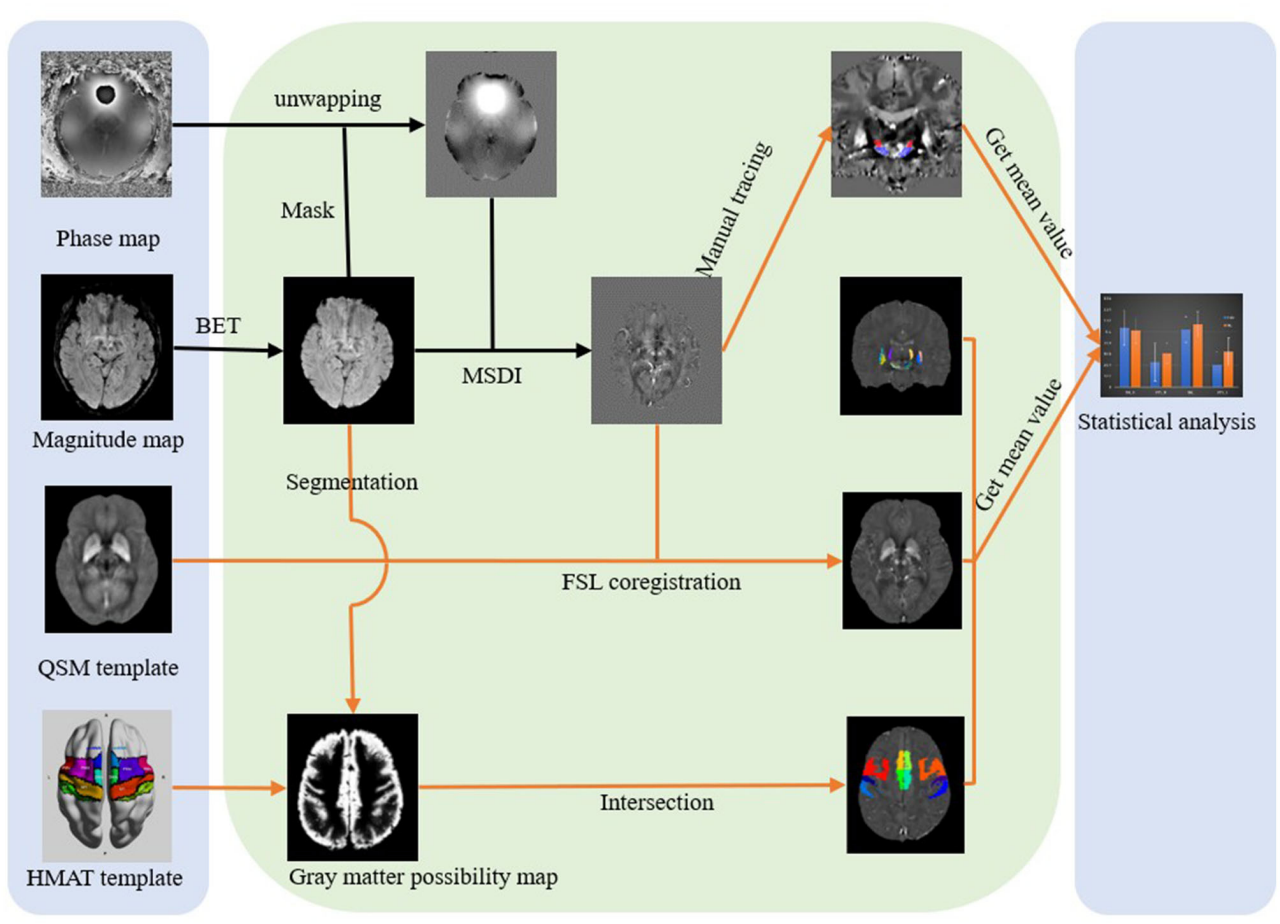
Data analysis flowchart. Three major modules from left to right are data acquisition, data processing, and statistical analysis. MSDI, multi-scale dipole inversion algorithm; FSL, FMRIB Software Library; HMAT, standard space motor cortex division human motor area template.

QSM image reconstruction was performed using the pipeline for multi-echo combined data provided in QSMbox (https://gitlab.com/acostaj/QSMbox), an open-access MATLAB toolkit. The processing steps were as described by Thomas et al. (28) (Figure 2) and specifically included (1) the phase data being unwrapped with a discrete Laplacian method based on the phase images; (2) the BET2 algorithm in FSL (https://fsl.fmrib.ox.ac.uk) being used to extract and mask the brain structure based on the magnitude images; (3) a background field suppression steps that included Laplacian boundary value extraction and spherical mean-value filtering; and (4) susceptibility maps estimating via the MSDI algorithm (29).

QSM spatial standardization was performed using FMRIB Software Library (FSL V6.0). Individual susceptibility maps were co-registered to spatially normalized QSM templates using affine and non-linear transformation (30).

### 2.4. Definition of regions of interest

The regions of interest (ROIs) of motor-related cortices in the motor CSTC loop were extracted from the gray matter part of the Human Motor Area Template (HMAT) (31). These included the primary motor cortex (M1) and primary somatosensory cortex (S1), which are located in the sensorimotor cortex (SMC). The pre-supplementary motor area (pre-SMA) and supplementary motor area proper (SMA proper) that are in the mesial premotor cortex (MPMC) were included. The other included locations were in the lateral premotor cortex (LPMC), which included the dorsal aspects of LPMC (PMd) and the ventral aspects of LPMC (PMv). To confine the analysis to the gray matter, individual magnitude images were first segmented, and then the gray matter probability maps were registered to the QSM standard space using affine and non-linear transformation. After registration, the intersections between the standardized gray matter probability map and the HMAT template were taken to define the gray matter part of the HMAT template.

Furthermore, the ROIs of subcortical nuclei in the motor CSTC loop were created based on the brain parcellation template that was previously constructed using QSM model data (30). These included the putamen (PUT), globus pallidus (GP), thalamus lateral nucleus (THAL_VL), substantia nigra pars reticulata (SNpr), and substantia nigra pars compacta (SNpc). In addition, the subthalamic nucleus (STN), a crucial node of the motor CSTC loop, was not contained in the aforementioned template and thus was manually traced in an individual susceptibility map using a 3D slicer (https://www.slicer.org/), as well as its nearby nucleus, the substantia nigra (SN).

### 2.5. Statistical analysis

For all these ROIs, mean QSM values were extracted separately for statistical analyses. To examine inter-group differences, chi-square tests were used for the analysis of gender distribution. Additionally, independent *t*-tests were used for indicators conforming to a normal distribution (one sample K–S test) or homogeneity of variance (Levene's test for homogeneity of variance) (such as age); otherwise, the Mann–Whitney U-test was used. Analysis of covariance (ANCOVA) was used for data conforming to a normal distribution and homogeneity of variance on regional susceptibility with age as a covariate; otherwise, a Mann–Whitney U-test was used. A partial correlation analysis was performed to explore the relationships between susceptibility indicators and each item of clinical manifestations, onset age, attack frequency, and attack duration included, while controlling for the effect of age. All statistical analyses were performed using SPSS 23.0 software (IBM Inc., Armonk, New York, USA). Data were considered statistically significant at the level of a *p*-value of < 0.05.

### 2.6. Sensitivity analysis

To address the effect of gray matter volume (GMV), we additionally performed ANCOVA on the mean QSM value for any ROI with age, total intracranial volume (TIV), and its GMV as covariates. TIV and GMV were estimated from T1-weighted images using the CAT12 (https://www.nitrc.org/projects/cat/) segmentation pipeline with mostly the default configurations except for the resolution configured at 1 mm. T1-weighted scans were acquired via a 3-dimensional magnetization prepared rapid gradient Echo (MPRAGE) sequence with the following parameters: TR = 2,110 ms, TE = 3.18 ms, FA = 9°, ST = 0.73 mm, FOV=232 × 232 mm^2^, matrix size = 320 × 320, NEX = 1; TA = 4 min, 36 s.

## 3. Results

### 3.1. General clinical information

As seen in Figure 1, two family members from the enrolled pedigree died decades ago, leaving only 23 members available for the current project. Of the nine PKD subjects, one was excluded for concurrent glioma. Of the 14 unaffected subjects, four declined to participate due to personal reasons. The general clinical information of both groups is summarized in Table 1. The PKD patients shared the same mutation of c.324_334del (p. Val109Argfs^*^21) in the second exon of the PRRT2 gene. Their onset age ranged from 8 to 10 years, and the attack generally lasted for <30 s with a frequency that varied from 0.3 to 120 times per month. There were no statistically significant differences between PKD and controls in age, gender, or mental health scores (*p* > 0.05).

**Table 1:** Analysis of general clinical information results.

**Characteristics**	**PKD group**	**NC group**	**Statistic (T)**	***p***
Age (year)	41.380 ± 19.777	48.600 ± 18.887	0.790	0.441
Gender (male/female)	4/4	5/5	Crosstab	1
Onset age (year)	9.500 ± 1.690	—		
Attack duration (s)	<30	—		
Attack frequency (per month)	0.3 to 120	—		
Symptom laterality (Right/Left/Both)	0/0/8	—		
SAS	42.750 ± 10.740	37.700 ± 6.977	1.207	0.245
SDS	51.000 ± 12.189	44.600 ± 11.501	1.143	0.270
SCL-90	18.803 ± 5.703	14.544 ± 2.142	U	0.078^b^

Continuous variables presented as mean ± standard deviation.

^b^Unequal variance by Levene's test.

### 3.2. Motor-related cortices

**Figure 3:**
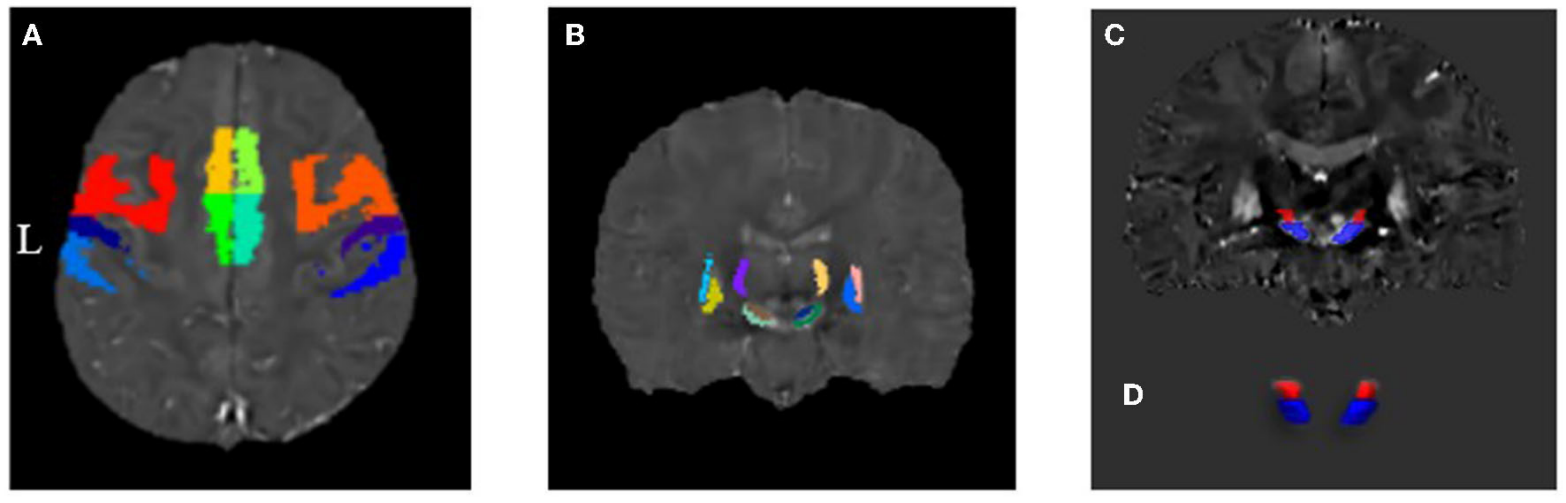
Distribution map of each region of interest in the motor CSTC loop. **(A)** Gray matter components of the motor-related cortices derived from the HMAT division template. **(B)** Motor-related subcortical nuclei derived from the QSM template by Chunlei Liu et al. **(C, D)** Coronal plane and three-dimensional (3D) representation of substantia nigra and subthalamic nucleus by manual segmentation.

The gray matter template for each motor cortex division in the HMAT template is shown in Figure 3A, and their corresponding mean susceptibility values are reported in Table 2. When controlling for age, the analysis of the bilateral M1 regions with unequal variance indicated that QSM values were significantly higher in PKD than in controls. Moreover, ANCOVA of other regions showed that those with PKD had decreased QSM values in the supplementary motor areas compared to controls while also having overall increased QSM values in S1 and PMd. In the PMv regions, PKD patients were found with significantly increased QSM values bilaterally when compared to controls (Table 2).

**Table 2:** Comparison of susceptibility part per million (ppm) in the motor-related cortices.

**Cerebral hemisphere**	**ROI**	**PKD group**	**NC group**	**Statistic (*F*)**	***p***	***p'***
Right side	M1	0.0021 ± 0.0012	−0.0003 ± 0.0043	U	0.003^*b^	0.169
	S1	0.0009 ± 0.0015	−0.0011 ± 0.0029	3.096	0.099	0.122
	SMA	0.0095 ± 0.0037	0.0116 ± 0.0037	2.377	0.144	0.270
	preSMA	0.0109 ± 0.0030	0.0147 ± 0.0045	4.402	0.053	0.067
	PMd	0.0007 ± 0.0020	−0.0010 ± 0.0027	2.006	0.177	0.204
	PMv	0.0032 ± 0.0018	−0.0002 ± 0.0026	9.394	0.008^*^	0.023^*^
Left side	M1	0.0019 ± 0.0018	−0.0022 ± 0.0048	U	0.000^*b^	0.053
	S1	−0.0001 ± 0.0031	−0.0008 ± 0.0029	0.238	0.633	0.657
	SMA	0.0102 ± 0.0023	0.0105 ± 0.0061	0.099	0.757	0.642
	preSMA	0.0149 ± 0.0030	0.0156 ± 0.0033	0.359	0.558	0.349
	PMd	0.0005 ± 0.0026	−0.0003 ± 0.0020	0.587	0.455	0.501
	PMv	0.0021 ± 0.0018	−0.0010 ± 0.0015	14.301	0.002^*^	0.004^*^

Continuous variables presented as mean ± standard deviation.

p, analysis with age as a covariate.

*p'*, analysis with age, TIV, and GMV as covariates.

^b^Unequal variance by Levene's test.

^*^Statistically significant at p < 0.05.

### 3.3. Motor-related subcortical nuclei

The distribution of each subcortical nucleus in the motor CSTC loop is shown in Figures 3B–D, and their corresponding mean susceptibility values are displayed in Table 3. When controlling for age, the PKD group showed significantly decreased QSM values in the bilateral SNpr regions compared to the controls. No statistically significant changes were observed in QSM values of other regions by ANCOVA (Table 3).

**Table 3:** Comparison of susceptibility (ppm) in motor-related subcortical nuclei.

**Cerebral hemisphere**	**ROI**	**PKD group**	**NC group**	**Statistic (*F*)**	***p***	***p'***
Right side	PUT	0.0345 ± 0.0144	0.0447 ± 0.0133	2.605	0.127	0.103
	GP	0.0807 ± 0.0215	0.0971 ± 0.0262	1.924	0.186	0.330
	THAL_VL	−0.0156 ± 0.0052	−0.0171 ± 0.0108	0.102	0.753	0.880
	SNpr	0.0485 ± 0.0443	0.0861 ± 0.0217	U	0.000^*b^	0.065
	SNpc	0.0622 ± 0.0319	0.0745 ± 0.0169	1.119	0.307	0.153
	SN (manual)	0.1066 ± 0.0306	0.0986 ± 0.0213	0.538	0.474	0.494
	STN (manual)	0.0448 ± 0.0341	0.0588 ± 0.0163	1.215	0.288	0.252
Left side	PUT	0.0254 ± 0.0119	0.0347 ± 0.0136	2.269	0.153	0.124
	GP	0.0899 ± 0.0201	0.1108 ± 0.0273	3.508	0.081	0.119
	THAL_VL	−0.0206 ± 0.0087	−0.0235 ± 0.0097	0.376	0.549	0.817
	SNpr	0.0132 ± 0.0339	0.0375 ± 0.0183	U	0.000^*b^	0.032^*^
	SNpc	0.0762 ± 0.0376	0.0877 ± 0.0205	0.638	0.437	0.413
	SN (manual)	0.1037 ± 0.0228	0.1086 ± 0.0205	0.180	0.678	0.640
	STN (manual)	0.0406 ± 0.0228	0.0577 ± 0.0242	2.332	0.148	0.122

Continuous variables represented as mean ± standard deviation.

*p*, analysis with age as a covariate.

*p'*, analysis with age, TIV, and GMV as covariates.

^b^Unequal variance by Levene's test.

^*^Statistically significant at p < 0.05.

### 3.4. Correlation analysis

No significant correlation was found in the present study between the various susceptibility indicators investigated and clinical manifestations including onset age, attack frequency, and attack duration (*p* > 0.05).

### 3.5. Sensitivity analysis

We performed a sensitivity analysis to further exclude the effects of TIV and GMV. The results were approximately the same for both the cortical and subcortical ROIs (Table 2). The susceptibilities in bilateral PMv and the left SNpr still had significant inter-group differences, and the susceptibilities in bilateral M1 and the right SNpr turned out to have tendencies for significant inter-group differences.

## 4. Discussion

Bioavailable iron is required for numerous biological processes in the brain, such as the synthesis of neurotransmitters, myelin formation, and neuronal energy metabolism, and thus is essential for maintaining normal neurological function (32). When iron homeostasis is disrupted, any excess levels may result in the release of reactive oxygen, increased oxidative stress, and cell death. Similarly, iron deficiency results in decreased activity of iron-dependent enzymes, leading to impaired development of brain structure and function (33). In addition to age-specific and region-specific requirements, diseases are among the important factors that disrupt iron homeostasis (33). Studies have demonstrated that neurological diseases with movement disorders, such as Parkinson's disease (19, 20), Huntington's disease (21), or epilepsy (22–25), may have altered iron homeostasis in the brain. In the study, we show for the first time the altered interictal iron levels in the brains of people with PKD.

PKD is an autosomal-dominant neurologic disorder with incomplete penetrance (34). The gene of PRRT2 is the first identified causative gene to the primary type of PKD (3). It accounts for one-third of PKD patients and 91% of familial PKD patients (35). The present study enrolled a big Chinese PKD pedigree whose affected members all shared the same c.324_334del (p. Val109Argfs^*^21) mutation of the PRRT2 gene. The pure genetic background shared by the PKD patients helped greatly to eliminate an important confounding factor of genotyping, making the interpretation of the results more credible.

Our results indicated that in the selected ROIs, participants in both groups exhibited the highest mean susceptibility values in GP followed by SNpc, while other subcortical nuclei had lower values, and the susceptibility values were lowest in motor-related cortices (Tables 1, 2). Previous studies demonstrated that QSM could measure iron content in the brain tissue (36), and the brain parenchyma has the highest levels in the extrapyramidal system, followed by the cortex, while the white matter has almost none (37). Therefore, our results followed the same pattern as previous findings, suggesting the high reliability of our data and data processing method.

Additionally, our results showed that the motor cortex gray matter components in PKD patients have significantly increased susceptibility values in the bilateral M1 and PMv regions. Since the anatomical contrast in the susceptibility maps of the human brain primarily comes from tissue iron (paramagnetic) and myelin (diamagnetic), the simultaneous presence or co-localization of these two complicates the interpretation of susceptibility changes. We hypothesized that the increased susceptibility of the gray matter motor cortex is likely a combined effect of two mechanisms. The first mechanism is increased iron content. Iron participates in the synthesis of neurotransmitters; thus, increased levels can enhance neurotransmitter synthesis capacity, increasing output functions in the brain. Studies have shown that the cortical medulla oblongata adjacent to the central sulcus is more prone to iron accumulation, which affects motor and decision-making signaling (38). A second mechanism is reduced astrocytes in the gray matter. Astrocytes are present in the gray matter (39), and their function includes controlling the number of synapses, clearing released neurotransmitters from synapses, regulating blood flow, and providing lactate energy to neurons (40, 41). Thus, we conjectured that a decline in astrocytes decreases their clearing capacity of released neurotransmitters, resulting in more excitatory glutamatergic neurotransmitters being transported to the basal ganglia. Noting that in the last decade, multiple attempts have been made to separate the contributions of paramagnetic and diamagnetic susceptibility sources for QSM. With the algorithms progressed from linear regression (39), three-pool complex signal modeling named DECOMPOSE-QSM (42) to the recently proposed comprehensive complex data modeling and an iterative voxel-specific magnitude decay kernel estimating algorithm named APART-QSM (43), more reliable susceptibility separation maps could be generated. It is believed that the maturation of susceptibility source separation technology will be helpful in validating our hypotheses about the underlying mechanisms.

The analysis of the subcortical nuclei involved in the motor CSTC loop found that PKD patients exhibited significantly lower susceptibility values in the bilateral SNpr regions. Previous studies reported that in deep gray matter structures such as subcortical nuclei, the effect of myelin is limited, and other paramagnetic metals are negligible; therefore, the susceptibility values can reliably be quantified as changes in iron content (36). Thus, the decline of SNpr region susceptibility values indicates lower levels of iron. The substantia nigra has an abundant iron-neuromelanin complex (37), a dark pigment that is structurally similar to melanin in the skin and the iris. It is produced through the oxidation of levodopa and dopamine and has a high affinity and chelating ability for iron and other metals, making it the main iron storage site in catecholaminergic ganglion (44). A decline in iron levels in the substantia nigra is inferred to be iron reallocation in the brain tissue as reported in premanifest Huntington's disease patients by van Bergen et al. (45). The SN has a large number of dopaminergic neurons with a continuous metabolic activity that is particularly susceptible to oxidative stress (46). Iron plays a key role in enhancing reactive oxygen species (ROS) and mitochondrial damage (47). Therefore, changes in iron in the substantia nigra can lead to functional changes in the substantia nigra and play an important role in the pathogenesis of motor dysfunction diseases (48).

We conducted sensitivity analyses to explore the effect of TIV and GMV. The magnetic susceptibility values of the bilateral PMv and the left SNpr were retained to be of significant group differences. This observation not only further supported the robustness of the alterations of susceptibility distribution but also suggested that the alterations were not caused by gray matter volume changes.

There are several limitations in this study. First, the sample size was relatively small, and the study was cross-sectional. It thus could not be determined whether the disruption in brain iron hemostasis is just an epiphenomenon of the pathological state or a catalyst of iron metabolism dysregulation promoting the development and progression of PKD. Therefore, longitudinal studies with larger sample sizes are required in future studies. Second, the participants were enrolled from a pedigree having c.324_334del (p. Val109Argfs^*^21) mutation in the second exon of the PRRT2 gene, which only accounts for most cases of PKD. Extreme caution should be exercised when generalizing the results across different families with different gene mutations. Third, voluntary movement control is regulated by the coordination of the pyramidal and extrapyramidal systems. Findings in PKD have shown susceptibility changes in both cortex and basal ganglia nuclei, but their respective roles in the disease are unknown and require further investigation. Moreover, our study lacked histopathological data validation. Histopathological data are also needed to determine whether brain iron levels can be used as a biological indicator of PKD.

## 5. Conclusion

We utilized QSM imaging technology for the first time to investigate brain iron levels in participants with familial PKD. Preliminary results showed that PKD patients had altered brain iron levels. In the cortical gray matter area involved with the motor CSTC loop, susceptibility values were generally elevated, especially in the bilateral M1 and PMv regions. In the subcortical nuclei regions involved with the motor CSTC loop, susceptibility values were generally lower, especially in the bilateral substantia nigra regions. Our results provide new evidence for the neuropathogenesis of PKD and suggest that an imbalance in brain iron levels may play a role in PKD. However, further histopathological investigations are needed to confirm the changes in brain iron levels and to provide more evidence for the changes as a neuroimaging marker for PKD.

## Data availability statement

The raw data supporting the conclusions of this article will be made available by the authors, without undue reservation.

## Ethics statement

The studies involving human participants were reviewed and approved by Ethics Review Committee from Xiangya Hospital of Central South University. The patients/participants provided their written informed consent to participate in this study.

## Author contributions

FX and TM contributed to data collection and manuscript preparation. JT, LZ, JG, and HL contributed to the manuscript review and editing. DW contributed to conceptualization, data analysis, manuscript review, and supervision. GZ contributed to supervision and funding acquisition. All authors contributed to the article and approved the submitted version.

## References

[1] Sanger TD Chen D Fehlings DL Hallett M Lang AE Mink JW. Definition and classification of hyperkinetic movements in childhood. Mov Disord. (2010) 25:1538–49. 10.1002/mds.23088 20589866 PMC2929378

[2] Silveira-Moriyama L Gardiner AR Meyer E King MD Smith M Rakshi K. Clinical features of childhood-onset paroxysmal kinesigenic dyskinesia with PRRT2 gene mutations. Dev Med Child Neurol. (2013) 55:327–34. 10.1111/dmcn.12056 23363396

[3] Chen WJ Lin Y Xiong ZQ Wei W Ni W Tan GH. Exome sequencing identifies truncating mutations in PRRT2 that cause paroxysmal kinesigenic dyskinesia. Nat Genet. (2011) 43:1252–U116. 10.1038/ng.1008 22101681

[4] Lu B Lou SS Xu RS Kong DL Wu RJ Zhang J. Cerebellar spreading depolarization mediates paroxysmal movement disorder. Cell Rep. (2021) 36:109743. 10.1016/j.celrep.2021.109743 34551285

[5] Graybiel AM Aosaki T Flaherty AW Kimura M. The basal ganglia and adaptive motor control. Science. (1994) 265:1826–31. 10.1126/science.8091209 8091209

[6] Shirane S Sasaki M Kogure D Matsuda H Hashimoto T. Increased ictal perfusion of the thalamus in paroxysmal kinesigenic dyskinesia. J Neurol Neurosurg Psychiatry. (2001) 71:408–10. 10.1136/jnnp.71.3.408 11511723 PMC1737540

[7] Liu W Xiao Y Zheng T Chen GX. Neural mechanisms of paroxysmal kinesigenic dyskinesia: insights from neuroimaging. J Neuroimaging. (2021) 31:272–6. 10.1111/jon.12811 33227178

[8] Kim JH Kim DW Kim JB Suh SI Koh SB. Thalamic involvement in paroxysmal kinesigenic dyskinesia: a combined structural and diffusion tensor MRI analysis. Hum Brain Mapp. (2015) 36:1429–41. 10.1002/hbm.22713 25504906 PMC6869556

[9] Zhou B Chen Q Gong Q Tang H Zhou D. The thalamic ultrastructural abnormalities in paroxysmal kinesigenic choreoathetosis: a diffusion tensor imaging study. J Neurol. (2010) 257:405–9. 10.1007/s00415-009-5334-9 20012544

[10] Long Z Xu Q Miao HH Yu Y Ding MP Chen H. Thalamocortical dysconnectivity in paroxysmal kinesigenic dyskinesia: combining functional magnetic resonance imaging and diffusion tensor imaging. Mov Disord. (2017) 32:592–600. 10.1002/mds.26905 28186667

[11] Zhou B Chen Q Zhang Q Chen L Gong Q Shang H. Hyperactive putamen in patients with paroxysmal kinesigenic choreoathetosis: a resting-state functional magnetic resonance imaging study. Mov Disord. (2010) 25:1226–31. 10.1002/mds.22967 20629125

[12] Luo C Chen Y Song W Chen Q Gong Q Shang HF. Altered intrinsic brain activity in patients with paroxysmal kinesigenic dyskinesia by PRRT2 mutation: altered brain activity by PRRT2 mutation. Neurol Sci. (2013) 34:1925–31. 10.1007/s10072-013-1408-7 23532549

[13] Zhang Y Ren J Qin Y Yang C Zhang T Gong Q. Altered topological organization of functional brain networks in drug-naive patients with paroxysmal kinesigenic dyskinesia. J Neurol Sci. (2020) 411:116702. 10.1016/j.jns.2020.116702 32058179

[14] Beard JL Connor JR. Iron status and neural functioning. Annu Rev Nutr. (2003) 23:41–58. 10.1146/annurev.nutr.23.020102.075739 12704220

[15] Tan GH Liu YY Wang L Li K Zhang ZQ Li HF. PRRT2 deficiency induces paroxysmal kinesigenic dyskinesia by regulating synaptic transmission in cerebellum. Cell Res. (2018) 28:90–110. 10.1038/cr.2017.128 29056747 PMC5752836

[16] Mills E Dong XP Wang F Xu H. Mechanisms of brain iron transport: insight into neurodegeneration and CNS disorders. Future Med Chem. (2010) 2:51–64. 10.4155/fmc.09.140 20161623 PMC2812924

[17] Wang Y Liu T. Quantitative susceptibility mapping (QSM): decoding MRI data for a tissue magnetic biomarker. Magn Reson Med. (2015) 73:82–101. 10.1002/mrm.25358 25044035 PMC4297605

[18] Eskreis-Winkler S Zhang Y Zhang J Liu Z Dimov A Gupta A. The clinical utility of QSM: disease diagnosis, medical management, and surgical planning. NMR Biomed. (2017) 30:e3668. 10.1002/nbm.3668 27906525

[19] Chen Q Chen Y Zhang Y Wang F Yu H Zhang C. Iron deposition in Parkinson's disease by quantitative susceptibility mapping. BMC Neurosci. (2019) 20:23. 10.1186/s12868-019-0505-9 31117957 PMC6532252

[20] Guan X Xuan M Gu Q Huang P Liu C Wang N. Regionally progressive accumulation of iron in Parkinson's disease as measured by quantitative susceptibility mapping. NMR Biomed. (2017) 30:e3489. 10.1002/nbm.3489 26853890 PMC4977211

[21] Dominguez JF Ng AC Poudel G Stout JC Churchyard A Chua P. Iron accumulation in the basal ganglia in Huntington's disease: cross-sectional data from the IMAGE-HD study. J Neurol Neurosurg Psychiatry. (2016) 87:545–9. 10.1136/jnnp-2014-310183 25952334

[22] Zimmer TS David B Broekaart DWM Schidlowski M Ruffolo G Korotkov A. Seizure-mediated iron accumulation and dysregulated iron metabolism after status epilepticus and in temporal lobe epilepsy. Acta Neuropathol. (2021) 142:729–59. 10.1007/s00401-021-02348-6 34292399 PMC8423709

[23] Kahn-Kirby AH Amagata A Maeder CI Mei JJ Sideris S Kosaka Y. Targeting ferroptosis: a novel therapeutic strategy for the treatment of mitochondrial disease-related epilepsy. PLoS ONE. (2019) 14:e0214250. 10.1371/journal.pone.0214250 30921410 PMC6438538

[24] Chen S Chen Y Zhang Y Kuang X Liu Y Guo M. Iron metabolism and ferroptosis in epilepsy. Front Neurosci. (2020) 14:601193. 10.3389/fnins.2020.601193 33424539 PMC7793792

[25] Cai Y Yang Z. Ferroptosis and its role in epilepsy. Front Cell Neurosci. (2021) 15:696889. 10.3389/fncel.2021.696889 34335189 PMC8319604

[26] He J Tang H Liu C Tan L Xiao W Xiao B. Novel PRRT2 gene variants identified in paroxysmal kinesigenic dyskinesia and benign familial infantile epilepsy in Chinese families. Exp Ther Med. (2021) 21:504. 10.3892/etm.2021.9935 33791013 PMC8005681

[27] Cao L Huang X Wang N Wu Z Zhang C Gu W. Recommendations for the diagnosis and treatment of paroxysmal kinesigenic dyskinesia: an expert consensus in China. Transl Neurodegener. (2021) 10:7. 10.1186/s40035-021-00231-8 33588936 PMC7885391

[28] Thomas GEC Leyland LA Schrag AE Lees AJ Acosta-Cabronero J Weil RS. Brain iron deposition is linked with cognitive severity in Parkinson's disease. J Neurol Neurosurg Psychiatry. (2020) 91:418–25. 10.1136/jnnp-2019-322042 32079673 PMC7147185

[29] Acosta-Cabronero J Milovic C Mattern H Tejos C Speck O Callaghan MF. A robust multi-scale approach to quantitative susceptibility mapping. Neuroimage. (2018) 183:7–24. 10.1016/j.neuroimage.2018.07.065 30075277 PMC6215336

[30] Zhang YY Wei HJ Cronin MJ He NY Yan FH Liu CL. Longitudinal atlas for normative human brain development and aging over the lifespan using quantitative susceptibility mapping. Neuroimage. (2018) 171:176–89. 10.1016/j.neuroimage.2018.01.008 29325780 PMC5857468

[31] Mayka MA Corcos DM Leurgans SE Vaillancourt DE. Three-dimensional locations and boundaries of motor and premotor cortices as defined by functional brain imaging: a meta-analysis. Neuroimage. (2006) 31:1453–74. 10.1016/j.neuroimage.2006.02.004 16571375 PMC2034289

[32] Thirupathi A Chang YZ. Brain iron metabolism and CNS diseases. Adv Exp Med Biol. (2019) 1173:1–19. 10.1007/978-981-13-9589-5_1 31456202

[33] Connor JR Menzies SL Burdo JR Boyer PJ. Iron and iron management proteins in neurobiology. Pediatr Neurol. (2001) 25:118–29. 10.1016/S0887-8994(01)00303-4 11551742

[34] Huang XJ Wang T Wang JL Liu XL Che XQ Li J. Paroxysmal kinesigenic dyskinesia: clinical and genetic analyses of 110 patients. Neurology. (2015) 85:1546–53. 10.1212/WNL.0000000000002079 26446061

[35] Tian WT Zhan FX Liu ZH Liu Z Liu Q Guo XN. TMEM151A variants cause paroxysmal kinesigenic dyskinesia: a large-sample study. Mov Disord. (2022) 37:545–52. 10.1002/mds.28865 34820915

[36] Langkammer C Schweser F Krebs N Deistung A Goessler W Scheurer E. Quantitative susceptibility mapping (QSM) as a means to measure brain iron? A post mortem validation study. Neuroimage. (2012) 62:1593–9. 10.1016/j.neuroimage.2012.05.049 22634862 PMC3413885

[37] Hallgren B Sourander P. The effect of age on the non-haemin iron in the human brain. J Neurochem. (1958) 3:41–51. 10.1111/j.1471-4159.1958.tb12607.x 13611557

[38] Acosta-Cabronero J Betts MJ Cardenas-Blanco A Yang S Nestor PJ. *In vivo* MRI mapping of brain iron deposition across the adult lifespan. J Neurosci. (2016) 36:364–74. 10.1523/JNEUROSCI.1907-15.2016 26758829 PMC4710766

[39] Stuber C Morawski M Schafer A Labadie C Wahnert M Leuze C. Myelin and iron concentration in the human brain: a quantitative study of MRI contrast. Neuroimage. (2014) 93:95–106. 10.1016/j.neuroimage.2014.02.026 24607447

[40] Allen NJ. Astrocyte regulation of synaptic behavior. Annu Rev Cell Dev Biol. (2014) 30:439–63. 10.1146/annurev-cellbio-100913-013053 25288116

[41] Lezmy J Arancibia-Carcamo IL Quintela-Lopez T Sherman DL Brophy PJ Attwell D. Astrocyte Ca(2+)-evoked ATP release regulates myelinated axon excitability and conduction speed. Science. (2021) 374:eabh2858. 10.1126/science.abh2858 34648330 PMC7611967

[42] Chen J Gong NJ Chaim KT Otaduy MCG Liu C. Decompose quantitative susceptibility mapping (QSM) to sub-voxel diamagnetic and paramagnetic components based on gradient-echo MRI data. Neuroimage. (2021) 242:118477. 10.1016/j.neuroimage.2021.118477 34403742 PMC8720043

[43] Li Z Feng R Liu Q Feng J Lao G Zhang M. APART-QSM: an improved sub-voxel quantitative susceptibility mapping for susceptibility source separation using an iterative data fitting method. Neuroimage. (2023) 274:120148. 10.1016/j.neuroimage.2023.120148 37127191

[44] Haining RL Achat-Mendes C. Neuromelanin, one of the most overlooked molecules in modern medicine, is not a spectator. Neural Regeneration Research. (2017) 12:372–5. 10.4103/1673-5374.202928 28469642 PMC5399705

[45] van Bergen JM Hua J Unschuld PG Lim IA Jones CK Margolis RL. Quantitative susceptibility mapping suggests altered brain iron in premanifest huntington disease. AJNR Am J Neuroradiol. (2016) 37:789–96. 10.3174/ajnr.A4617 26680466 PMC4867278

[46] Snyder AM Connor JR. Iron, the substantia nigra and related neurological disorders. Biochim Biophys Acta. (2009) 1790:606–14. 10.1016/j.bbagen.2008.08.005 18778755

[47] Walker T Michaelides C Ekonomou A Geraki K Parkes HG Suessmilch M. Dissociation between iron accumulation and ferritin upregulation in the aged substantia nigra: attenuation by dietary restriction. Aging. (2016) 8:2488–508. 10.18632/aging.101069 27743512 PMC5115902

[48] Garcia-Malo C Novo-Ponte S Castro-Villacanas Farzamnia A Boi S Miranda Castillo C Romero Peralta S. Correlation between systemic iron parameters and substantia nigra iron stores in restless legs syndrome. Sleep Med. (2021) 85:191–5. 10.1016/j.sleep.2021.07.027 34343769

